# Clinical outcomes of mandibular body fracture management using wire-reinforced intraoral composite splints in 15 cats

**DOI:** 10.3389/fvets.2025.1552682

**Published:** 2025-03-24

**Authors:** Joanna Pakula, Alix Freeman, Andrew Perry

**Affiliations:** Department of Dentistry, Oral and Maxillofacial Surgery, Eastcott Veterinary Referrals, Part of Linnaeus Veterinary Limited, Swindon, United Kingdom

**Keywords:** splint, fracture, mandibular body, composite, wire, minimally invasive, occlusion

## Abstract

The study assesses the use of wire-reinforced intraoral composite splints (WRICS) for stabilising mandibular body fractures in feline patients. It reviews 15 cases treated at a referral centre, focusing on the effectiveness of WRICS in achieving stable fracture repair, occlusion, and patient comfort. The fractures were most commonly between the canine tooth and third premolar (73%). Results indicate that WRICS can provide effective stabilisation with a median healing time of 8 weeks. Normocclusion was achieved in 14 out of 15 cases. Major complications were found in two cases (13%) and were associated with soft tissue ulceration. This study supports WRICS as a minimally invasive, reliable approach to mandibular body fracture stabilisation in cats.

## 1 Introduction

Maxillofacial fractures in cats are a common consequence of traumatic events such as traffic accidents (RTA), high-rise syndrome or fights (1, 2). A recent study (3) showed that mandibular fractures are a common component of maxillofacial trauma cases—in the cohort of 45 cats, 86.7% had a mandibular fracture. Based on the same study, the region most frequently involved was the symphysis/parasymphyseal region affecting 64.1% of cats. Rostral mandibular body and mid/caudal mandibular body fractures were present in 17.8 and 15.6%, respectively.

Fracture morphology is influenced by both the underlying aetiology and patient-specific factors, as well as the characteristics of the applied force, including its direction, rate and magnitude (4). The anatomical location and configuration of these fractures are critical in determining appropriate treatment methods and can assist in predicting the outcome (5, 6). Plate fixation can provide bridging support for comminuted fractures or those with gaps resulting from bone or tooth loss (7). This technique offers precise fracture reduction to reestablish anatomical relationships, effective fracture fixation to ensure stability, facilitates an early and safe return to function, simplifies the maintenance of patient airway and oral hygiene, and enhances nutrition. Non-invasive fracture repair management employs closed fracture stabilisation techniques, relying on the restoration of normal occlusion and radiographic evaluation to confirm proper alignment and apposition of fracture fragments (7). Non-invasive repair techniques should not be the first choice for highly comminuted fractures; the open reduction and internal fixation is a more accurate method in these cases (7, 8). However, fracture repair in the rostral and mid-mandibular region presents the following challenges (8).

- Tooth roots occupy a large volume of bone, which reduces the space available for screws to be placed, especially in the rostral mandible. It is crucial to avoid causing iatrogenic damage to tooth roots when using any fixation device. Penetration of the tooth root can cause loss of root substance, interruption of pulpal blood supply, root fractures, and can disrupt its blood supply, which will likely lead to pulp necrosis, periodontitis and tooth resorption. Furthermore, screw penetration creates a tract that can provide bacterial access, resulting in infection and, subsequently, a periapical lesion. In previous studies assessing the outcomes of screw penetration of tooth roots, a variety of lesions involving the periodontal ligament, dentine, cementum, pulp, and periapical tissue were noted (9, 10).
- Neurovascular structures in the mandibular canals may further limit screw or implant placement. In human maxillofacial surgery, the concept of “safe zones” to avoid nerve injury is documented in the literature (11). The mandibular canal, which contains the inferior alveolar nerve, restricts the vertical height of the safe zone in the mandibular body and angle. In human literature, the prevalence of inferior alveolar nerve (IAN) injury after fracture treatment using open reduction and internal fixation (ORIF) ranges from 0.4 to 91.3% (12). Patients complain of sensory disturbances such as pain, paraesthesia, dysaesthesia, hypoesthesia or anaesthesia involving the chin, lower lip and gums. In the study (11), the IAN and mental nerve (MN) neurosensory status worsened in 13% of patients who underwent mandibular fracture repair. It was found that fracture displacement, operator inexperience and two plate fixation were associated with a statistically significant risk for postoperative deterioration of IAN/MN sensation (11). In one study (13), unilateral paraesthesia of the lower lip and chin was observed in 7 out of 67 patients. CBCT analysis revealed a significant distance of 3.02 mm between the inferior alveolar nerve canal and the screws. It is hypothesised that concern with regard to the neurovascular bundle within the mandibular canal is not warranted in comminuted fractures of the angle and the body of the mandible as the fracture has compromised this structure (14). Further research is necessary to evaluate the complications associated with intracanal screw placement and its potential consequences in dogs and cats.

All the above limits the possibility of using ORIF treatment methods for mid-body and rostral mandibular fractures in dentate patients. In dogs, rostral and mid-body mandibular fractures can be reduced effectively with wire-reinforced intraoral splint techniques (WRICS) (15). The tooth crowns are integrated into fixation devices where there are some periodontally healthy teeth which can be used for anchorage. Various patterns of interdental wiring have been described, including Stout multi-loop (7), crossover (16), and modified Risdon (7). These techniques can be described as either non-invasive or minimally invasive. They capitalise on the biological principles of bone healing through closed reduction. Intraosseous and extraosseous vascularisation are both known to play major roles in mandibular bone healing (%^17^–^19^). Interdental splints are placed on the tension surface of the mandible (alveolar margin), and because fixation devices are strongest in tension (all stresses acting parallel to the longitudinal axis of the implant), it supports the basic biomechanical principle of tension-band fixation (20). One or more cerclage wires can be incorporated into interdental wires in edentulous regions of the mandible. This does not exposure the fracture site during surgery but does disrupt soft tissues more than conventional intraoral wire and composite splint. Where cerclage wires are incorporated, this technique is better described as ‘minimally’ invasive rather than non-invasive.

Fracture stabilisation using wire-reinforced intraoral splints provides efficient stabilisation and accurate anatomical reduction in canine patients (15). Rapidly restoring normal occlusion enhances recovery, limits soft tissue trauma, reduces pain, supports physiological jaw motion, allows expression of normal teeth function, and is one of the goals of maxillofacial trauma repair. Malocclusion has been implicated in the aetiology of some joint disorders such as degenerative temporomandibular joint disease in humans (21) and dogs (22).

Interdental wiring can avoid the need to disrupt the fracture hematoma and does not usually disrupt the periosteum or otherwise induce surgical trauma associated with more invasive repair techniques (7). Creating intraoral splints for dogs using dental composites has proven to be a safe, strong, and clinically effective alternative to open reduction and internal fixation (7, 15). Despite this, there is a paucity of evidence documenting the use and outcomes of WRICS in feline mandibular fractures. The objective of this study was to report the outcomes of WRICS for the treatment of mandibular body fractures in cats.

## 2 Materials and methods

### 2.1 Study population and data collection

Medical records of cats presented to the Dentistry and Oromaxillofacial Surgery Department at Eastcott Referrals, Swindon, UK, part of Linnaeus Veterinary Limited, between 2016 and 2024 were reviewed. Procedures were performed by European Veterinary Dental College diplomates and residents under supervision. Inclusion criteria were as follows: the presence of a mandibular body fracture, treatment with an intraoral wire-reinforced splint, and the availability of pre-operative and follow-up CT scan performed a minimum of 6 weeks following treatment. Data collected for each patient included signalment, body weight, trauma aetiology, fracture type, concurrent injuries, time elapsed from injury to repair with WRICS, wiring technique, teeth included in the splint, antibiotic use, time for bone healing, imaging modalities, and complications. Outcomes and complications were assessed from clinical data and computed tomography follow-up examinations.

### 2.2 CT imaging and anaesthesia

CT scans were performed using a Lightspeed 4 CT scanner (GE Healthcare) with a kVp of 120 and auto-mA, obtaining transverse, 0.625 mm collimated images. For intraoral dental radiographs, CR7 (iM3, Ireland) and the Vet Exam Pro software was used. All CT scans were conducted under general anaesthesia, and an anaesthetic plan was tailored by an anaesthesia specialist according to the cat’s clinical status. Anaesthesia maintenance involved intubation and administration of isoflurane in oxygen. CT images were assessed by a specialist and resident in dentistry and oral surgery. For most patients, WRICS were performed immediately following the CT scan, during the same anaesthetic.

### 2.3 Surgical procedure and fracture evaluation

Patients were positioned in lateral or sternal recumbency with the maxilla supported by a table-top mouth gag (IM3, Ireland). The oral cavity was rinsed with a 0.12% chlorhexidine solution (Hexarinse; Virbac). Teeth were cleaned using a piezoelectric scaler and evaluated clinically and radiographically to determine if those in the fracture line required treatment. Extraction of a tooth in a fracture line was performed if the tooth was luxated, had periodontitis or root fractures. Timing of dental treatment (initial vs. delayed), both of teeth within the fracture line, and teeth affected by unrelated oral pathology, was determined based on the severity of dental pathology. Pre- operative occlusion was assessed prior to intubation. Instability leading to malocclusion and inability to fully close the mouth was described as open occlusion. Oral and cutaneous wounds, if present, were cleaned, debrided if necessary, and surgically closed with resorbable monofilament suture material (Monocryl 5–0, Ethicon Inc., Somerville, NJ, USA). A comminuted fracture was characterised as a fracture involving three or more bone fragments. However, “minute” fragments were disregarded unless the entire bone or area was reduced to multiple small or micro fragments (23).

Fractures were reduced in a closed fashion to restore the occlusion of the teeth and the alignment of bony segments. As described in the study (15), occlusion was monitored peri-operatively by either closing the mouth over the endotracheal tube for cats with sufficient crown length to allow at least 50% interdigitation of the maxillary and mandibular canine teeth, or by performing a transmylohyoid intubation to allow complete closure of the mouth. Data relating to placement or use of a feeding tube was recorded for patients with inappetence or multiple skull fractures.

### 2.4 Fracture stabilisation

**Figure 1:**
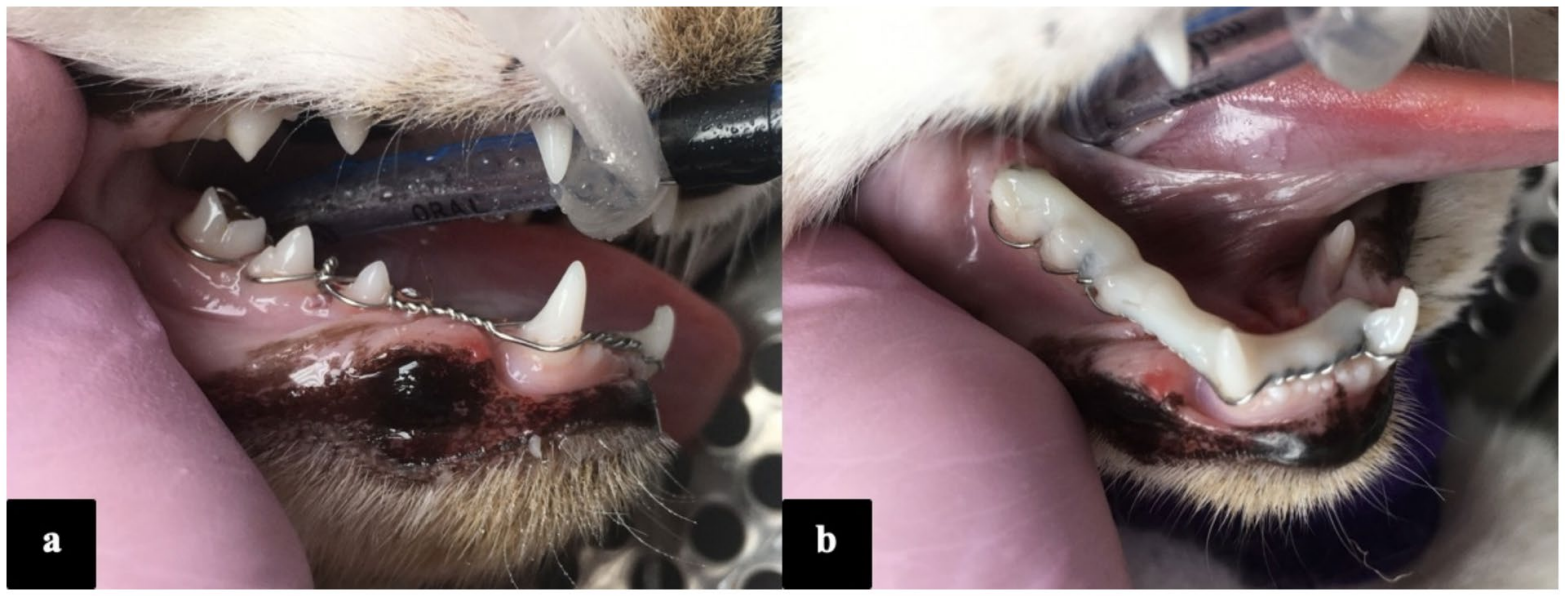
Photographs of the oral cavity taken under general anaesthesia, illustrating the stout-loop wiring technique **(a)** and the splint construction reinforced with a light-cured flowable composite **(b)**.

Fractures were stabilised using 24 or 26G wire placed in configurations based on the fracture conformation, most commonly between the mandibular first molar tooth and the contralateral canine tooth (Figure 1) or from the mandibular first molar tooth to the contralateral mandibular first molar tooth. Various wiring techniques were employed, including simple continuous (crossover (16)) interdental wiring, Stout loop wiring (7), modified Risdon wiring (7), and a vestibular arch bar. Initial wire placement was facilitated by using composite buttons on the distal aspect of the mandibular first molar and mesial aspect of the mandibular canine teeth as well as other teeth if needed, to prevent wire slippage during tightening. The interdental wiring bridged the fracture line, providing primary stabilisation until the composite was applied. In some patients with edentulous regions of the mandible, a cerclage wire was incorporated around the mandible and into the composite splint to help secure the splint in place without relying on tooth anchorage. In one patient, an appropriately sized metal endodontic file was inserted into the root canal of a canine root fragment and incorporated to anchor the wire structure. A metal endodontic file was inserted through the crown fracture site following total pulpotomy, initial root canal shaping, and irrigation with 5.25% sodium hypochlorite.

**Figure 2:**
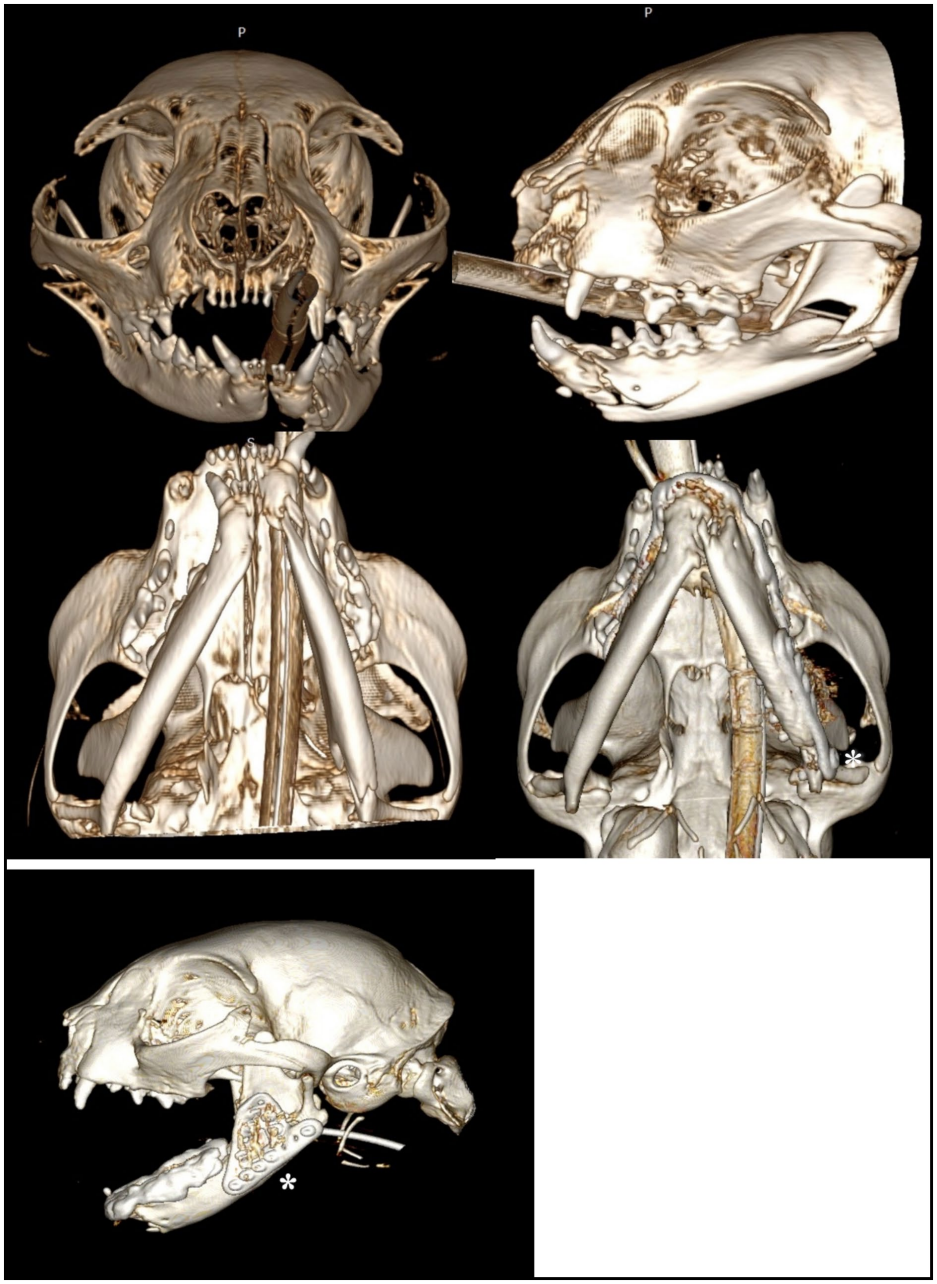
A 3D rendering of head computed tomography scans illustrating Case 8 (pre-operative and post-operative), in which WRICS treatment was performed concurrently with osteosynthesis using a ramus anatomical plate (RAP) to repair a caudal ramus fracture (asterisk).

**Figure 3:**
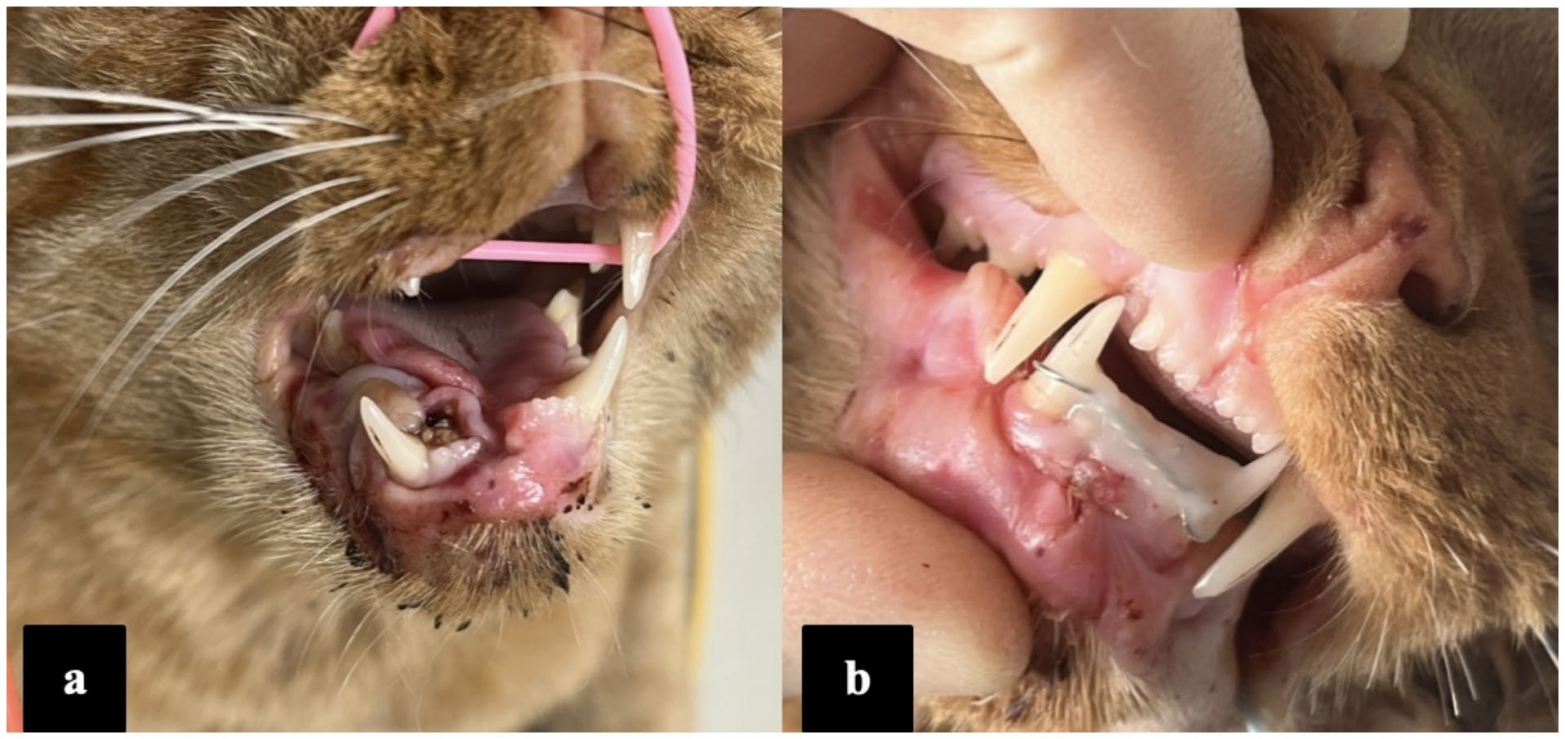
Photographs of the patient (case 3) showing preoperative **(a)** and immediate postoperative occlusion **(b)**.

Once the interdental wiring had been applied, the tooth surfaces were acid-etched for 20–30 s (phosphoric acid 38% etching gel, IM3, Ireland), rinsed, and air-dried. Following etching, the surface of the teeth were spot bonded with a 5th generation adhesive (Prime and Bond, Dentsply Sirona) and light-cured. A flowable composite (Grand Flow, Voco) or bis-acylic (Protemp 4 Garant, 3M ESPE) was applied directly onto the wire and tooth crowns, set with a curing light (for flowable composite), and shaped and polished to remove sharp edges. Patients with concurrent injuries received additional treatment or stabilisation as needed (Figure 2). Occlusion was checked intraoperatively and post-operatively (Figure 3).

### 2.5 Postoperative care

Cats with open fractures received antibiotics intraoperatively (potentiated amoxicillin 20 mg/kg intravenously) or/and postoperatively (amoxicillin-clavulanic acid 12.5–20 mg/kg given orally twice daily; up to 14 days, depended on the extend of concurrent facial fractures and soft tissue injuries). Patients received a combination of an opioid, a non-steroidal anti-inflammatory (NSAID), and/or gabapentin (5 mg/kg every 8 h), while hospitalised. Additional analgesia, duration of analgesia and type of NSAID was based on individual patient requirements. Patients who required nutritional support were fed via an oesophagostomy tube until they were voluntarily consuming their full resting energy requirements. Owners were advised to keep the cats indoors, offer only soft food, and restrict toys and chewing on hard objects while the WRICS was in place.

### 2.6 Complications and follow-up

Complications were classified as major if they jeopardised return to function (e.g., loosening of the WRICS, fracture instability, anorexia caused by the splint) and required additional general anaesthesia to evaluate the complication. Minor complications included oral inflammation and mild malocclusions that did not interfere with normal jaw closure or occlusal function and thus could be treated conservatively with medications such as pain relief.

**Figure 4:**
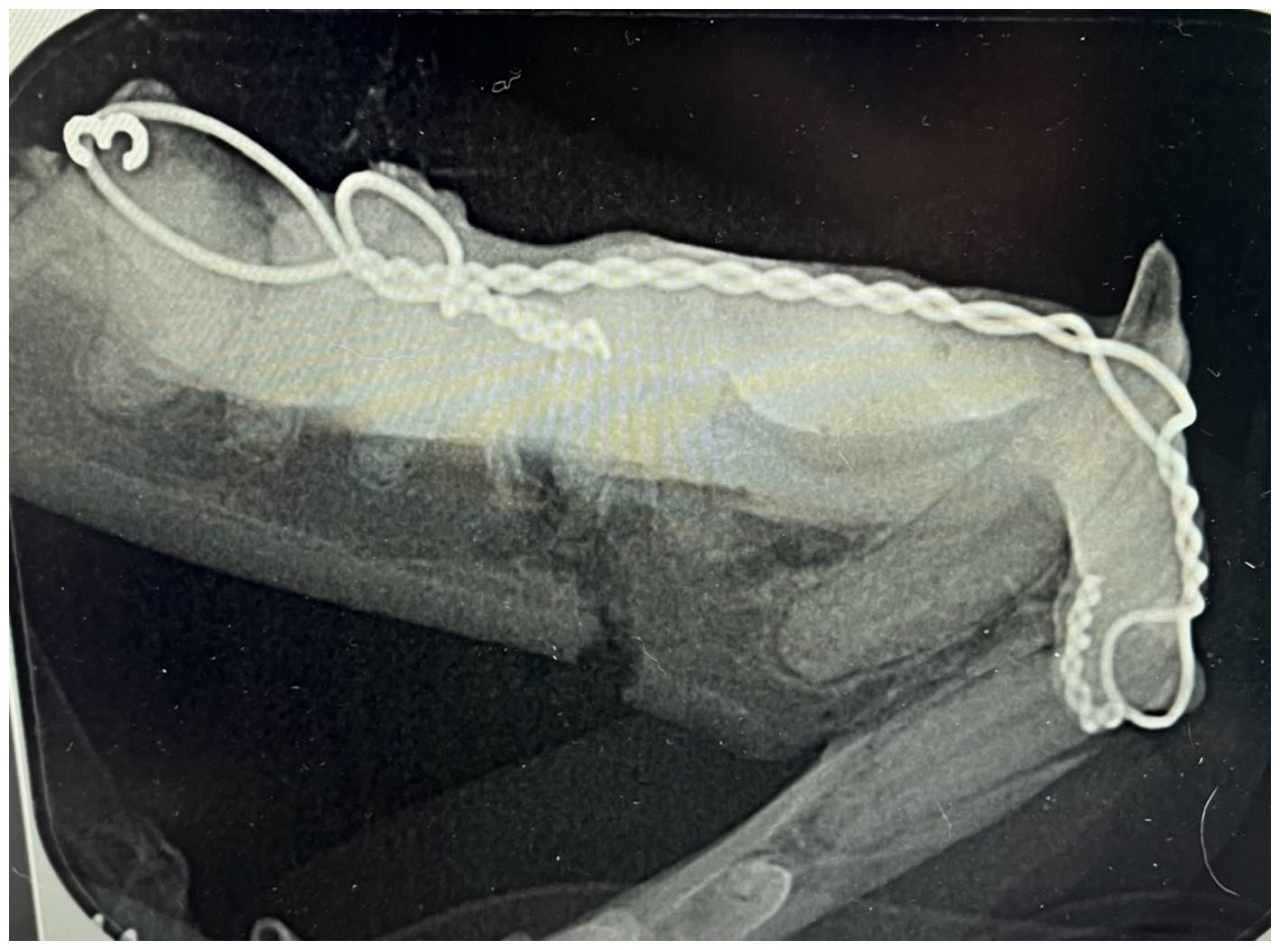
Immediate postoperative intraoral dental radiograph showing WRICS placement. These also reveals incomplete reduction of the ventral mandibular cortex (case 1).

Owners were advised to schedule the first postoperative CT scan at 6–10 weeks. All cats had pre and post-operative CT scan to assess healing. Dental radiography was used as an adjunctive imaging modality, for example where teeth were extracted at the time of splint removal (Figure 4). Radiographs themself were not used to assess bone healing. The images were evaluated for radiographic evidence of fracture healing (increased bone mineral density and bridging callus across the fracture lines) using MPR mode, high resolution head. If images lacked evidence of bone healing, patients were rescheduled for an additional re-check 3–4 weeks later. The timing of splint removal was determined by the attending clinician based on clinical stability and CT scan assessment of bone bridging the fracture site. Occlusion was evaluated after splint removal, and additional procedures were performed if necessary during that time, such as the extraction of teeth causing traumatic occlusion or teeth with untreated dental pathology which had been used to provide anchorage for the WRICS.

### 2.7 Data analysis

Categorical data are summarised by number and percentage, and continuous data by median and range. It was considered that the sample size was too small for reliable statistical analysis. However, multiple regression of time to eat on age, weight and time until presentation was fitted with backwards elimination of nonsignificant terms. A similar regression of time to bone healing was fitted to age, weight, time to presentation and time to eating. Results were compared to those from nonparametric regression. Analysis was undertaken in Minitab 21 and R 4.3.3. Significance was taken as *p* < 0.05.

## 3 Results

Fifteen client-owned cats were identified in the medical records database that met the inclusion criteria. The cohort comprised 9 neutered males (60%), and 6 neutered females (40%) with a mean age of 79 months (range 33–183 months). The cats’ body weights ranged from 3.2 kg to 7 kg, with a median weight of 4.3 kg at the time of admission.

The breed distribution included nine domestic shorthairs, one domestic longhair, one Burmese, one Siamese, one Chausie, one Bengal, and one Exotic shorthair.

The causes of trauma were identified as follows: nine unknown causes (60%), three road traffic accidents (20%), dog bites (1), falls from height (1), and home accidents due to impact from a fence panel (1). All patients were referred for primary fracture management.

The median time from trauma to WRICS application was 2 days (range <1 to 8 days). Fractures were categorised as open orally in seven cases (47%), open orally with a cutaneous wound in five cases (33%), closed in two cases (13%), and a cutaneous wound only in one case. There was no information in the clinical records for one patient.

**Figure 5:**
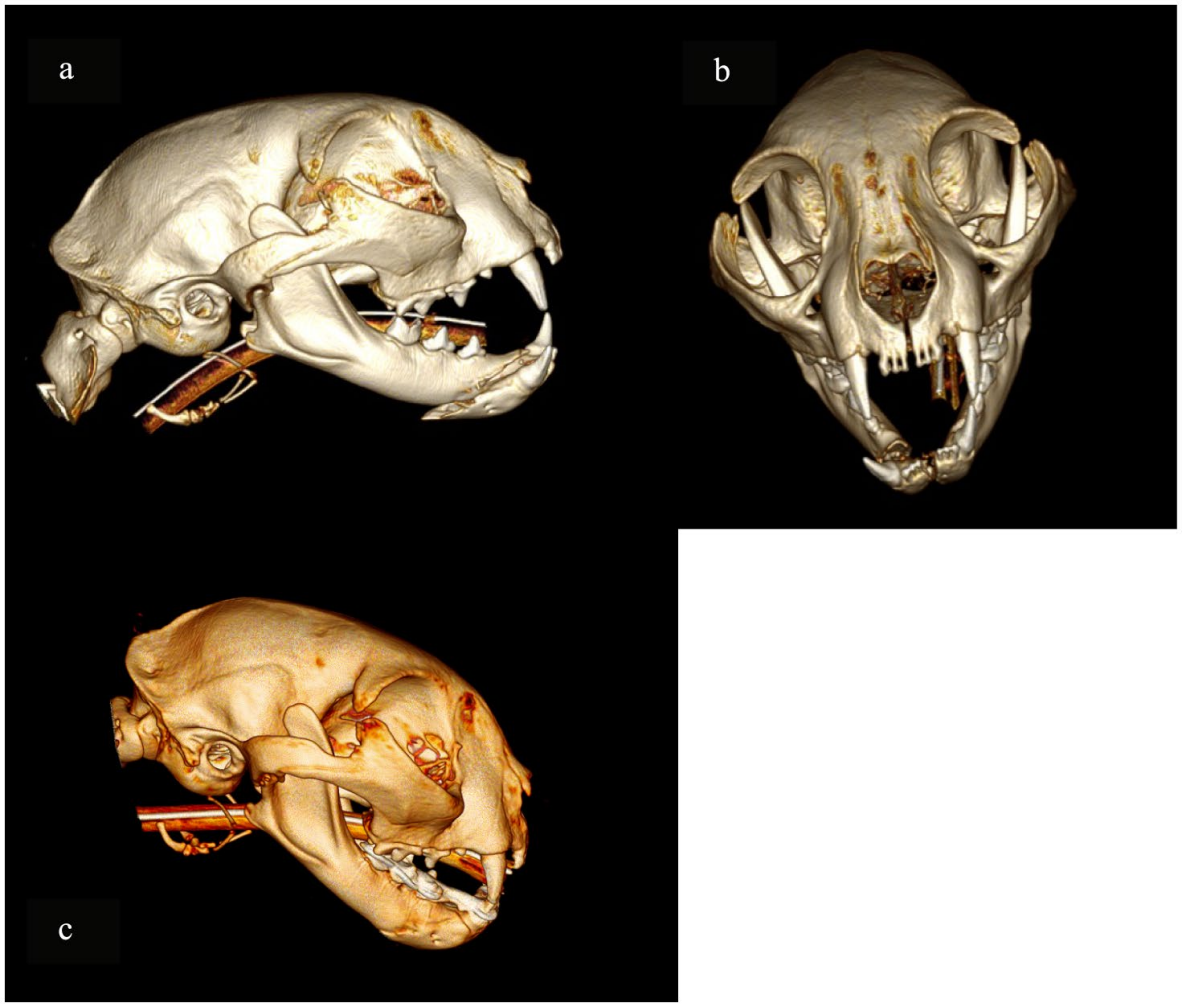
A 3D rendering of head computed tomography scans depicting the most common fracture configuration observed in the studied group of cats: a complete, oblique fracture of the mandibular body located between the mandibular canine tooth and the third premolar **(a,b)** Case 3. **(c)** Postoperative CT, 3D rendering image of the skull demonstrates the fracture site at the time of WRICS removal.

The fracture locations were most commonly present between the canine tooth and third premolar (73%) and between the third and fourth premolar (27%, Figure 5).

Concurrent symphyseal separation was present in five cases (33%). Additional fractures included a caudal mandibular fracture in three cats, a parasagittal fracture of the mandibular condylar head in one cat, and multiple mid-facial fractures in two cases. Eleven fractures were simple and complete (73%), three were comminuted (20%), and one had a defect.

In total, tooth involvement in the fracture line included the left mandibular third premolar tooth in three cases, the right mandibular third premolar in four cases, the right mandibular third incisor tooth in one case, the right mandibular canine tooth in three cases and the left mandibular canine tooth in one case.

Intubation methods were recorded for all 15 cats: 11 were intubated transorally (73%) and 4 underwent transmylohyoid endotracheal intubation (27%).

Eight cats (53%) had feeding tubes placed during treatment. One cat had a nasal tube placed 24 h prior to referral, one cat had an oesophageal feeding tube placed prior to referral, one cat had an oesophageal feeding tube placed during a re-check due to anorexia, and five cats (33%) had oesophageal feeding tubes placed on the day of the procedure at our hospital. Time to resume eating varied from immediately post-surgery to 6 weeks (in the cat with maxillomandibular fixation). For one patient who had an oesophageal feeding tube placed (case 13), time to resume eating was not recorded. Excluding cats who were also treated with maxillomandibular fixation (MMF) (case 14, case 12) and the cat for which time to resume eating was not available (case 13), the median time between WRICS placement and time to the resumption of eating in the remaining cats was 2 days (range 0–28 days).

Antibiotics were used in 13 cats: two cats had perioperative intravenous injection of amoxicillin and antibiotics were discontinued afterwards, 10 cats had a course of amoxicillin and clavulanic acid (varied 5–14 days and discontinued), and one cat had an antibiotic course started 2 days after WRICS placement due to post-operative complications (sublingual swelling, wound dehiscence and oral ulcer).

The configuration of interdental wiring included modified Risdon interdental wiring in eight cases (53%), a modified Stout loop in five cases (33%), an interdental crossover in one case, and a vestibular arch bar in one case. In one patient, an appropriately sized metal endodontic file was inserted into the root canal of a canine root fragment and incorporated to anchor the wire structure.

The major anchorage points were between the first mandibular molar and contralateral canine in 12 cases (80%) and involving all mandibular teeth in three cases (20%).

Three cats also required surgical management of other facial fractures. One cat had an ipsilateral caudal mandibular fracture stabilised with RAP (ramus anatomical plate) and two cats had maxillo-mandibular fixation (MMF) incorporated into WRICS during the treatment. In two cats, cerclage wires were used to provide additional anchorage for the WRICS.

**Figure 6:**
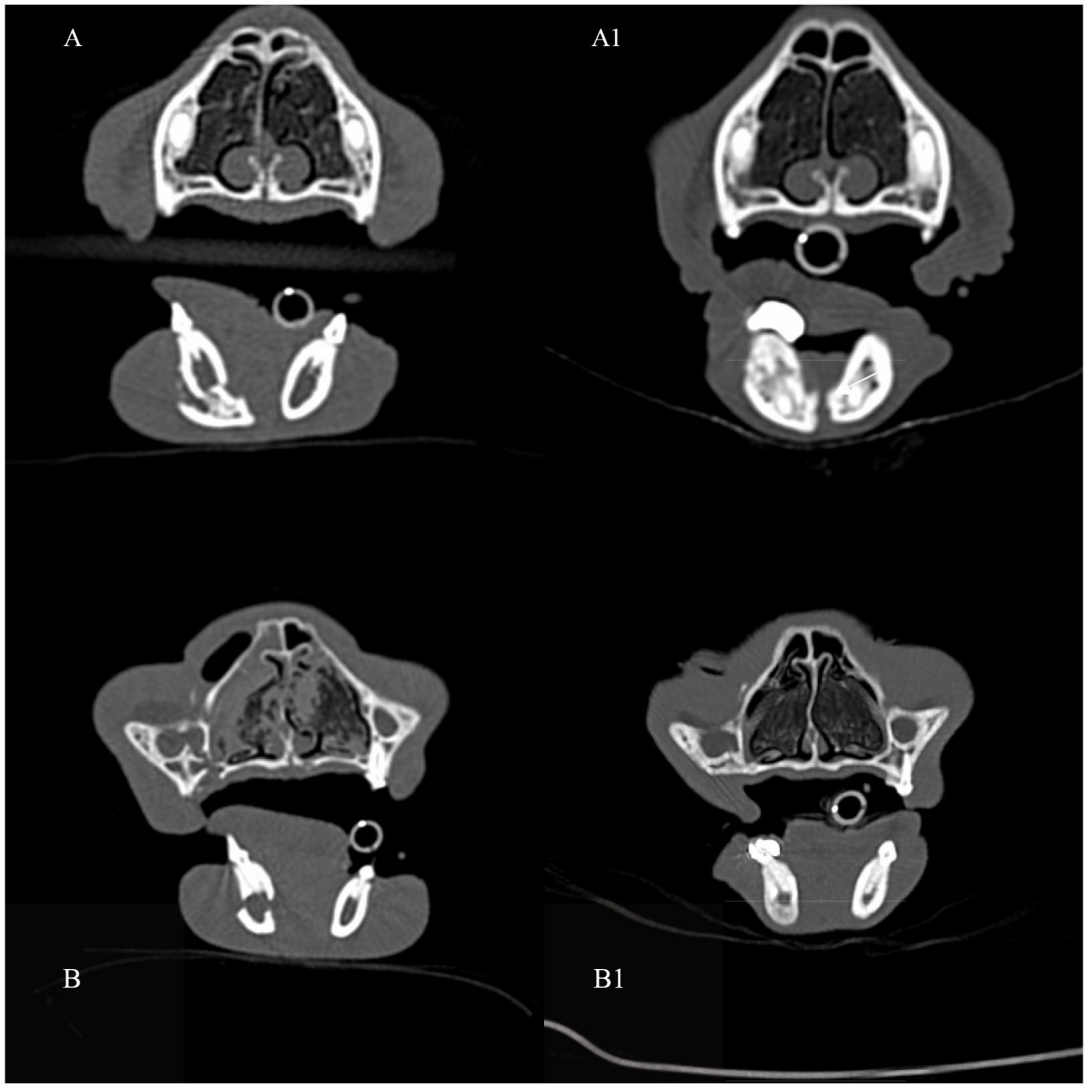
Transverse computed tomography (CT) images in bone algorithm demonstrating examples of pre-operative mandibular body fractures **(A,B)** and post-operative progression of bone healing (arrows), at the time of WRICS removal **(A1,B1)**.

Post-operative checks were scheduled between 2 and 14 days after surgery. The duration of the WRICS application ranged from 6 to 10 weeks, with a median time of 8 weeks (Figure 6). One patient exhibited callus formation at the fracture site 6 weeks post-operatively, which was stable but not fully united, allowing for splint removal.

**Figure 7:**
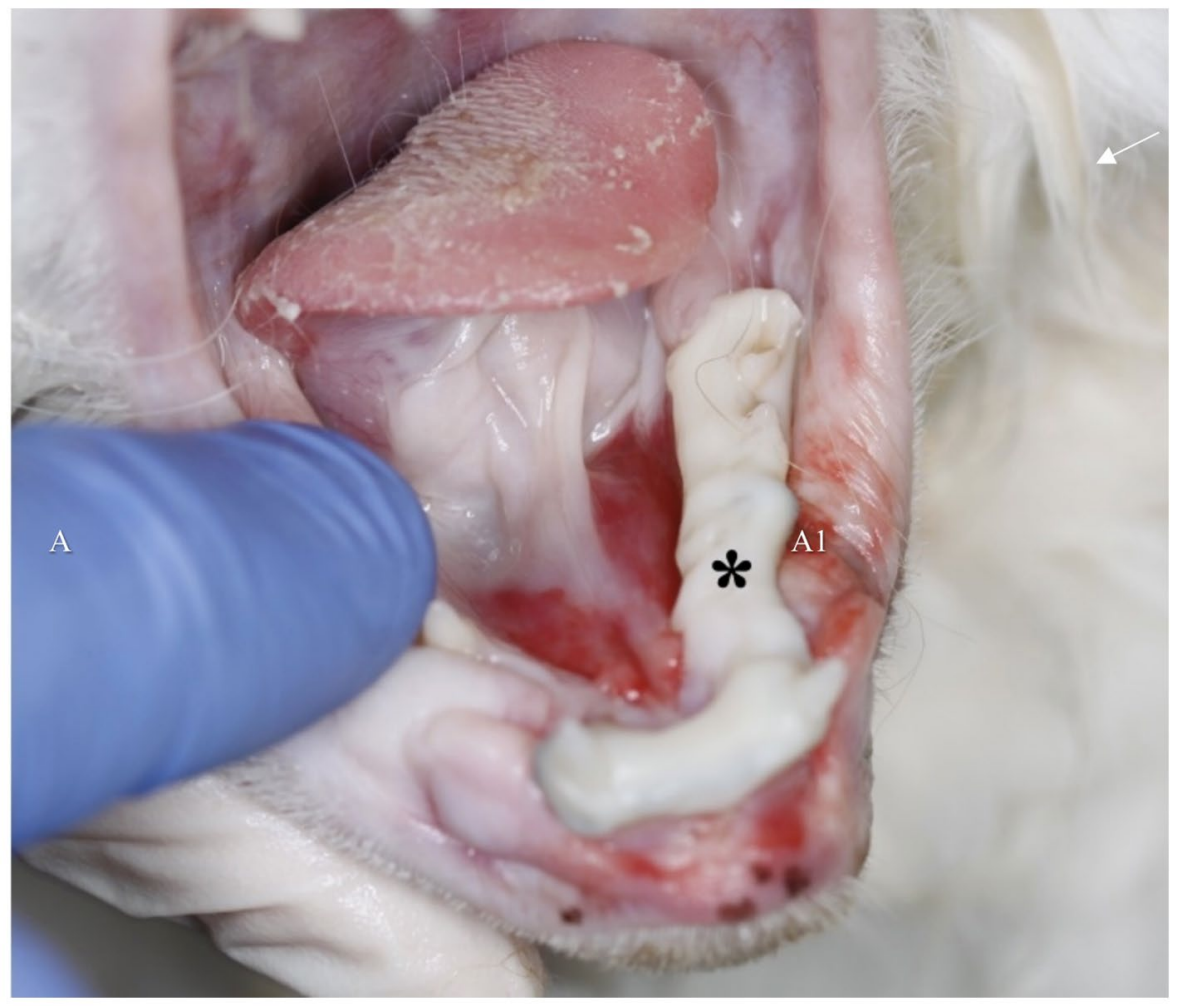
Sharp edges of the intraoral splint may lead to soft tissue and sublingual ulceration, which contributed to postoperative anorexia and pain in two patients (asterisk). Following splint remodelling under general anaesthesia, the anorexia and soft tissue irritation resolved.

Three of the 15 cases experienced major complications while the splint was *in situ*. Two patients (13%) developed lingual ulceration due to sharp fragments of the WRICS within the first 7 days of treatment (Figure 7), and one patient experienced MMF failure 2 weeks post-operatively after jumping from a radiator, but WRICS remained intact. MMF was replaced.

Minor complications occurred in four cases (27%), including mild ptyalism (possibly related to intravenous opioid administration) in one case, pyrexia in one case, mild oral mucosal indentation from a mandibular canine tooth in one case, and signs of pain in one case.

Pre-operative occlusion was described as open occlusion in 14 cases (93%), and not recorded in one case.

Post-operative occlusion after WRICS removal was reported as normocclusion in 14 cases (93%), and traumatic contact between the maxillary 4th premolar and mandibular 1st molar with inability to fully close the mouth in one case. One case was unable to completely close the mouth immediately after MMF removal but had developed a normal occlusion by the time of the re-check CT scan and splint removal 4–6 weeks later without any further intervention.

In total, 14 out of 15 cats had a normocclusion, which required no further treatment. Only one of the 14 patients had a malocclusion following splint removal, which required further treatment. A single tooth extraction in this patient allowed normal mouth closure. Seven patients (47%) underwent extractions during WRICS removal due to periodontal disease or tooth resorption. One of these patients required tooth extraction of the mandibular canine within the fracture line due to the development of periodontal disease.

The multiple regression of time to eat did not reveal any significant predictors. However, the multiple regression of time to bone healing with backwards elimination revealed two significant predictors, namely weight (*p* = 0.004) and time until presentation (*p* = 0.024). Both terms were negative, suggesting quicker recovery in heavier cats and those with a delayed presentation. Nonparametric regression broadly supported these conclusions (*p* = 0.017 and *p* = 0.052 respectively).

## 4 Discussion

This retrospective study is the first case series to describe the clinical outcomes of wire-reinforced intraoral composite splints to manage mandibular body fractures in cats. Our results demonstrated that WRICS can be successfully implemented as the sole method of mandibular body fracture repair in felines. The study confirmed radiographically complete bone healing in all cases in a median time of 8 weeks. We report our clinical experience, as well as both clinical and radiological outcomes.

The median time between fracture occurrence and application of the WRICS was 2 days, ranging from 1 to 8 days. Prompt stabilisation of fractures is critical to minimise pain, prevent further injury, and facilitate early return to function. The variation in the time to application highlights potential delays in referral or treatment initiation, which could be attributed to factors such as patient suitability for general anaesthesia following a severe head trauma episode, owner delay or logistical challenges in accessing specialised care.

In this cohort, 53% of the cats required the placement of feeding tubes. The decision to place feeding tubes in five cats (33%) on the day of the procedure shows that a proactive approach may need to be taken to ensure nutritional support in the immediate post-operative period, particularly in cases where oral intake was anticipated to be compromised, either as a result of the complexity of the injuries, patient temperament, or for patients who were already hyporexic or anorexic at presentation. The variability in time to resume eating, ranging from immediately post-surgery to up to 4 weeks, particularly in the case involving maxillomandibular fixation, underscores the complexity of managing these cases post-operatively. Excluding cases with MMF and the one case with missing information, the median time between WRICS removal and the resumption of eating was 2 days. Nutritional support should be considered for animals with oral intake disruptions exceeding 5 days. Specific maxillofacial indications for nutritional intervention may include prolonged mandibular immobilisation, suboptimal fracture stabilisation and multiple fractures. Postoperative inappetence is particularly prevalent in cats undergoing maxillofacial trauma repair or surgical procedures involving the nasal cavity, and these patients derive significant benefit from enteral nutritional support (24). Based on the authors’ clinical experience, oesophageal feeding tube placement is recommended for cats with concomitant multiple facial or cranial fractures, fractures involving the nasal cavity, pterygoid bone, pterygiod hamulus, as well as those presenting with extensive ocular trauma or an inability to prehend food preoperatively despite appropriate analgesia. Feeding tube placement can be performed under general anaesthesia during initial diagnostic imaging to facilitate early nutritional support prior to definitive fracture stabilisation. Conversely, cats that are eating comfortably before fracture repair, particularly those with isolated mandibular fractures, may not require enteral feeding support.

Fractures were predominantly open orally (80%), which underscores the risk of contamination and subsequent infection. The presence of cutaneous wounds in conjunction with oral mucosal wounds in 27% of cases further complicates the clinical scenario, necessitating meticulous wound management. Perioperative and post-operative systemic antibiotics are indicated as the majority of these patients have open fractures (7). In retrospective study on mandibular fractures in dogs, antibiotics were administered to 7 1 of 105 dogs (68%) (25). In dogs treated with antibiotics, osteomyelitis, malunion, and delayed union were significantly less common (25). In humans, mandibular fractures exhibit a higher incidence of infection compared to maxillary fractures (26). Antibiotic prescribing practices for postoperative mandibular fractures in humans show significant variation. Some authors advocate for prophylactic perioperative antibiotic administration, commencing before surgery and limited to a duration of no more than 24 h (27). Two human studies have demonstrated that postoperative oral antibiotic administration for 5–10 days does not confer any additional benefit beyond perioperative intravenous antibiotic administration in patients with mandibular fractures (28, 29). However, in cases of mandibular fractures that communicate with the oral cavity, five randomised human studies indicate that antibiotic administration significantly reduces the risk of infection at the fracture site (27).

Four cats in this study had concomitant lip avulsion, which can be challenging to repair with a high risk of post-operative wound dehiscence (29). In two patients presented in the study, avulsion was repaired before referral and, in one case, resulted in dehiscence and exposure of the rostral bone fragment, which was later removed due to necrosis, resulting in a minor bone defect. Overall 13 out of 15 cats were given antibiotics empirically, as bacterial culture and sensitivity were not performed in any of the cases, and antibiotic use was justified based on clinical bone exposure and the severity of soft tissue injuries.

Because of the small number of cats included in the study, we cannot draw any conclusions related to fracture configuration and suitability for this method of fixation. Simple and complete fractures were predominant in the study and 14 out of 15 cats were presented with pre-operative malocclusion and inability to close the mouth. Independent of the type of fracture (comminuted, simple and complete, incomplete, or defect), treatment using WRICS resulted in normocclusion in 14 out of 15 cats. Three cats had other fixation devices placed in addition to the WRICS due to the severity of their injuries and the presence of concomitant caudal mandibular fractures: two cats had maxillo-mandibular fixation (case 12 and 14) and one cat was treated with a RAP plate to stabilise a unilateral caudal fracture (patient 8), which shows that WRICS can be successfully combined with other methods of fracture repair to provide stability and support for bone healing. Notably, only one case (case 12) failed to restore normocclusion, which involved the use of the cross-over technique. It is worth noting that this patient was diagnosed with multiple facial fractures, including a fracture of the left zygomatic process of the squamous temporal bone and a comminuted right caudal mandibular fracture. As post-operative imaging confirmed the accurate reduction of the rostral mandibular fracture with the splint, it is therefore likely that the upper facial fractures contributed more significantly to the post-operative malocclusion. Bone healing was confirmed on the follow-up CT scan when WRICS was removed. The time of the splint removal was based on previous (15), average fracture healing time was 2.37 months. The forementioned study provided data on healing time; however, the re-examination period for the patients was not clearly specified. Two figures—1.9 and 2.3 months—were reported as justification for the treatment duration. Consequently, follow-up imaging was scheduled within this timeframe. The timing of follow-up assessments was frequently influenced by individual client circumstances.

In our study, 36% of cats in the study had concurrent symphyseal separation. The most common fracture location was between the canine tooth and the third premolar, accounting for 79% of the cases. Less commonly, fractures were observed between the third premolar and first mandibular molar, accounting for 21% of the cases. It is important to note that a significant portion of the mandibular body in cats is occupied by tooth roots, particularly in the region of the mandible rostral to the third premolar. In total, seven third mandibular premolars and four mandibular canines were present in fracture lines within the cats in this cohort. If teeth were periodontally healthy and did not have increased mobility, they were incorporated into WRICS and decision about extraction or root canal treatment were made at the time of WRICS removal based on radiographic and clinical evaluation.

The duration of WRICS application varied between 6 and 10 weeks, with a median time of 8 weeks. One patient demonstrated callus formation at the fracture site 6 weeks post-operatively, which, while stable, had not yet fully united. Intraoral splints provide stabilisation for mandibular fractures but exhibit lower stiffness than intact mandibles, making primary bone healing unlikely, which was shown in the recent study (30). Despite the incomplete union, the decision to remove the splint at this stage suggests that the WRICS had provided sufficient stabilisation to support the early stages of healing and that the patient could continue to recover without the splint. Based on the author’s experience and the results of this study, the optimal duration for WRICS application is suggested to be between 8 and 10 weeks. According to the clinical notes for all cases included in the study, no iatrogenic damage to the teeth occurred in any patient during WRICS removal.

Among the major complications, two cases (13%) developed lingual ulceration due to sharp fragments of the WRICS within the first week post-treatment. The two cases that developed oral ulceration were re-presented due to ptyalism and hyporexia. In both cases, this was the result of sharp edges of composite caudally, on the lingual aspect of the splint. It is particularly challenging to create a smooth surface over the caudolingual aspect of the splint due to the small patient size, access to the caudal oral cavity and the presence of concurrent sublingual swelling in these head trauma patients. Adjustment of the splint, including the removal of sharp edges under general anaesthesia, resolved these issues, leading to improved patient comfort and a rapid increase in appetite. This complication underscores the importance of splint design to minimise soft tissue injury.

The occurrence of device failure (MMF) in one patient (case 14) 2 weeks post-operatively following an incident involving the patient jumping from a radiator indicates a potential vulnerability of the composite splints to external forces. In this case, WRICS remained intact, only MMF required replacement. A recent cadaveric study reported a 25% failure rate of intraoral splints due to adhesive failure (30). Wire instability, likely caused by challenges in securing it tightly around the tooth neck and adapting it to the surface, may have compromised bis-acryl composite bonding (30). Additionally, the limited dental surface for attachment further restricts intraoral splint efficacy in cats, highlighting its technical challenges (30). In the same study (30) 50% of intraoral splints failed due to fractures of the bis-acryl composite, which was not observed in present study. Furthermore, the previously cited cadaveric study demonstrated that the Risdon splint exhibited significantly greater deflection compared to intact mandibles (30). This finding may indicate that this wiring technique could be a consideration when selecting the optimal stabilisation method. Nevertheless, the instance of device failure highlights the importance of advising strict activity restriction during the healing phase to prevent complications that could jeopardise the integrity of the repair.

Minor complications were observed in 27% of the cases, with mild ptyalism potentially related to intravenous opioid administration being the most benign. Pyrexia in one case and signs of pain in another suggest that some level of systemic response or discomfort is to be expected. While mild ptyalism may not directly relate to the WRICS, it could indicate discomfort or irritation in the oral cavity, the general condition of the patient, the stress associated with hospitalisation, or the medications used. However, these complications were all manageable and did not appear to jeopardise the overall outcome.

The retrospective nature of the study has limitations such as the potential for under-reporting of complications, reliance on observations, and inability to standardise follow-up. Statistical analysis was performed; however, due to the small sample size and the limited number of cats in contrasting conditions, meaningful conclusions could not be drawn. The statistician noted that comparisons, such as three cases of major complications against the remaining 12 animals, were insufficiently powered to yield reliable results. The statistical findings should be interpreted with caution. Specifically, the observed association suggesting accelerated bone healing in heavier cats and those with delayed presentation may not accurately reflect biological reality. As discussed earlier, the timing of the follow-up CT scan was determined based on prior studies investigating the use of WRICS in dogs, representing an estimated average duration required for the formation of a stable bony bridge sufficient for splint removal. Furthermore, variability in follow-up timing was introduced as some pet owners opted for later-than-recommended imaging due to personal constraints.

The results of this study highlight both the potential and the challenges, of using minimally invasive wire-reinforced splints (WRICS) for mid-body and rostral mandibular fracture repair in feline patients. With a major complication rate of 13%, refinements in the technique and design of the splint may be necessary. Additionally, stringent post-operative care and monitoring are essential to mitigate the risk of complications, particularly those related to the fit and stability of the device. Further studies with a larger sample size would be beneficial in determining the long-term efficacy and safety of this approach.

## Data Availability

The original contributions presented in the study are included in the article/Supplementary material, further inquiries can be directed to the corresponding author.
