# Supplementary material for: Clinical outcomes of mandibular body fracture management using wire-reinforced intraoral composite splints in 15 cats
Source: Front Vet Sci. 2025 Mar 24;12:1552682. doi: 10.3389/fvets.2025.1552682 (PMC11973384; doi:10.3389/fvets.2025.1552682)
Supplement: Supplementary file 1 [file Table_1.docx]

|  | **Case 1** | **Case 2** | **Case 3** | **Case 4** | **Case 5** | **Case 6** | **Case 7** | **Case 8** | **Case 9** | **Case 10** | **Case 11** | **Case 12** | **Case 13** | **Case 14** | **Case 15** |
| --- | --- | --- | --- | --- | --- | --- | --- | --- | --- | --- | --- | --- | --- | --- | --- |
| **Breed** | DSH | DSH | DSH | Burmese | Siamese | DSH | DSH | DSH | DSH | Chausie | DSH | Bengal | DLH | Exotic shorthair | DSH |
| **Age (months)** | 33 | 96 | 56 | 183 | 99 | 116 | 87 | 89 | 47 | 43 | 138 | 36 | 86 | 51 | 36 |
| **Gender** | Female Neutered | Male neutered | Male neutered | Female neutered | Male neutered | Male neutered | Female neutered | Female neutered | Female neutered | Female neutered | Male neutered | Male neutered | Male neutered | Male neutered | Male neutered |
| **Weight** | 3.38kg | 7kg | 4.5kg | 3.43kg | 3.28kg | 3.9kg | 4.27kg | 3.55kg | 3.2kg | 4.44kg | 5.2kg | 4.92kg | 6kg | 4.38kg | 3.6kg |
| **Etiology** | High-rise | Unknown | Unknown | Unknown | Fence panel fell on him | Unknown | Dog bite | RTA | Unknown | Unknown | RTA | Unknown | Unknown | Unknown | RTA |
| **Pre-operative occlusion** | Open mouth | Not noted | Open mouth | Open mouth | Open mouth | Open mouth | Open mouth | Open mouth | Open mouth | Open mouth | Open mouth | Open mouth | Open mouth | Open mouth | Open mouth |
| **Post-operative occlusion** | Norm | Norm | Norm | Norm | Norm | Norm | Norm | Norm | Norm | Norm | Norm | Mal | Norm | After MMF removal: Mal; Norm on the last follow-up | Norm |
| **Time elapsed between fracture and WRICS application** | 3 days | <1 day | 1 day | 3 days | 2 days | 1-2 days | 2 days | 7-8 days | 2 days | 7 days | 2 days | 8 days | 3 days | 2 days | 2 days |
| **Wiring technique** | Modified Risdon | Unknown | Modified Risdon | Unknown | Modified Risdon | Modified Risdon | Modified Risdon | Modified Risdon | Modified Risdon | Modified stout loop | Unknown | Crossover | Arch bar + endodontic file | Modified stout-loop | Modified Risdon |
| **Fracture type** | Complete | Complete | Complete | Complete | Comminuted, complete | Complete | Complete, comminuted | Complete/defect/comminuted | Complete | Complete | Complete, minimally displaced | Complete | Complete, comminuted | Complete | Complete |
| **Location** | 407/408 | 407/408 | 404 | 404 | 304/307 | 307/308 | 404 | 304 | 404/407 | 304/307 | 304/307 | 304 | 404 | 304 | 407/408 |
| **Concurrent symphyseal separation** | No | No | Yes | No | No | No | No | Yes | No | No | No | Yes | Yes | Yes | No |
| **Skin / oral mucosa involvement** | No | Open orally | Open orally | Open orally | No | Open orally | Open orally | Open orally and open skin (lip avulsion) | Open orally | Open orally and open skin. Multiple metallic shards were present in the wound | Open orally | No information given | Open skin, open orally (lip avulsion) | Skin open, open orally (lip avulsion), | Skin open (lip avulsion), open orally |
| **Major complications** | None | None | None | None | Sublingual ulcer | Oral soft tissue ulcer | None | None | None | None | None | None | None | MMF failed and had to be replaced | None |
| **Minor complications** | None | None | None | Ptyalism | Pyrexia | None | None | Mild indentation, resolved without intervention | None | None | None | None | None | Pain | None |
| **Time to eat after WRICS placed** | 1 day | 1 day | Immediately post-op | 2 days | 4 days | 12 days | 2 days | 4 days | 1 day | 1 day | 4 days | 42 days | Not known | 28 days; after MMF removal | Immediately post-op |
| **Teeth in the fracture line** | 407 | 407 | 407, 404 | 404 | 304, 307 | None | None | None | None | None | 307 | None | 404, 403 | 307 | 407 |
| **Extractions because of post-operative malocclusion** | No | No | No | No | No | No | No | No | No | No | No | XSS 308,  309 | No | No | No |
| **Time for bone healing (weeks)** | 8 | 6 | 8 | 9 | 9 | 10 | 8 | 7 | 10 | 8 | 9 | 6 | 9 | 7 | 10 |
| **Intubation technique** | TMH | OT | TMH | OT | OT | TMH | OT | OT | OT | TMH | OT | OT | OT | OT | OT |
| **Other stabilisation methods** |  |  |  |  |  |  |  | RAP |  |  |  | MMF |  | MMF |  |
| **Oesophageal feeding tube** | No | No | No | No | Yes | Placed on a re-check | No | Yes | Yes | No | Yes | Yes | Yes | Yes | No |

TMH - transmylohyoid intubation

OT - orotracheal intubation

RAP - ramus anatomical plate

XSS - extraction

MMF - maxillomandibular fixation

Mal - malocclusion

Norm - normocclusion
